# Acceptability and Satisfaction of Eat My ABCs: A Mindful Eating Program for Preschoolers in Low-Income Families

**DOI:** 10.3390/nu18071103

**Published:** 2026-03-30

**Authors:** Hannah Lalonde, Esra’a Sawalmeh, Reese Buhlman, Sophia Tadavich, Yingcen Xie, Jiying Ling

**Affiliations:** 1 College of Nursing, Michigan State University, East Lansing, MI 48824, USA; sawalmeh@msu.edu (E.S.); buhlmanr@msu.edu (R.B.); tadavic1@msu.edu (S.T.); xieyingc@msu.edu (Y.X.); 2 School of Nursing and Rehabilitation, Shandong University, Jinan 250012, China

**Keywords:** mindful eating, acceptability, satisfaction, low socioeconomic status, low income, preschool, obesity, food insecurity, family, mindfulness

## Abstract

Background: Mindful eating strategies have shown promise in addressing obesity and food insecurity among children. However, limited research has examined the acceptability of mindful eating programs among low-socioeconomic families in rural areas. This study evaluated caregivers’ and teachers’ acceptability and satisfaction with a 14-week, school-based mindful eating program for preschoolers to inform future implementation. The program effectively improved home eating environment, household food insecurity, and child body mass index. Methods: A convergent parallel mixed methods study design was used to evaluate the 14-week mindful eating intervention among 200 preschoolers from 26 Head Start classrooms in rural Michigan, United States. After intervention completion, acceptability and satisfaction data were collected and assessed from 192 caregivers and 23 classroom teachers via (1) quantitative online survey data analyzed using descriptive statistics, and (2) qualitative data from individual interviews completed with a subset of parents and teachers that were analyzed with thematic analysis. Results: Both quantitative and qualitative data showed high acceptability and satisfaction. Caregivers (survey: 88.9%, interview: 94.1%) and teachers (survey: 87.0%, interview: 100%) were satisfied with the program. Teachers (survey: 82.6%, interview: 87.5%) agreed the program improved children’s eating behaviors. Most surveyed caregivers (81.1%) agreed the completion of child letters sent home after the program session helped caregiver–child communication. Several barriers and suggestions for implementation were also identified by interviewed teachers and caregivers, including the limited availability of specific fruits and vegetables in local areas, concerns about preschoolers’ comprehension of curriculum, and recommendations on improving recruitment strategies. Conclusions: This study highlighted the high acceptability and satisfaction of a mindful eating program among caregivers and classroom teachers. The findings offered implications for future interventions to integrate mindful eating programs into early childcare organizations to help address child obesity and food insecurity. Future research exploring nutrition-related policies to sustain implementation of mindful eating programs is needed. Clinical Trial Registration: The clinical trial was registered at ClinicalTrials.gov (NCT05780008) on 27 February 2023.

## 1. Introduction

Many young children and their families from low socioeconomic backgrounds face the dual challenges of childhood obesity and household food insecurity [1,2,3]. In the United States (U.S.), the prevalence of obesity among children aged 2–5 years was 12.7% in 2017–2020, with higher rates observed among children from low-socioeconomic families [4]. Furthermore, among U.S. children aged 2–11, the prevalence of obesity has steadily increased since 2011, reaching 20% overall and 14.9% among children aged 2–5 years in 2021–2023 [5,6]. Food insecurity affected 12.8% of U.S. households with children in 2022, but this rate was significantly higher, 27.6%, among households with incomes below the federal poverty threshold [7]. In addition, a longitudinal study of 28,353 children aged 2–5 years found that children living in households with food insecurity had 1.2 times higher odds of developing obesity than their food secure counterparts [8]. Preschoolers from socioeconomically disadvantaged households may have limited access to healthy food options and safe spaces for physical activity [9,10]. Additionally, food-insecure households often rely on energy-dense, nutrient-poor foods, contributing to poor dietary quality and a heightened risk of obesity [11,12,13]. This cyclical relationship underscores the urgent need for interventions that simultaneously increase access to nutritious foods and foster healthy eating habits from an early age.

Integrating mindful eating strategies into early childhood nutrition programs presents a potential solution to address both obesity and food insecurity concurrently. Mindful eating is an evidence-based approach that promotes awareness of hunger and satiety cues, enhances enjoyment of food, and reduces impulsive or emotional eating [14]. Research has shown that mindful eating can improve dietary behaviors, promote self-regulation, reduce emotional eating, and reduce obesity in both children and adults [15,16,17]. For instance, one meta-analysis of 10 studies reported that mindful eating interventions in adults had a significant effect on weight loss compared to non-intervention controls (effect size = −0.35) [18]. Similarly, another review of 15 studies identified that mindful eating techniques were associated with some positive improvements in reducing body mass index (BMI) and improving healthy eating behaviors [19]. However, research on mindful eating interventions specifically targeting preschoolers is very sparse. Furthermore, no studies to date have examined the direct impact of mindful eating intervention on reducing household food insecurity. Notably, one clinical trial with 117 women experiencing food insecurity found that mindful eating training enhanced short-term survival strategies, such as choosing immediately available smaller amounts of money and foods rather than delayed larger amounts, which may contribute to temporary reductions in food insecurity [20]. These findings highlight the potential of mindful eating to promote healthy weight and support food-related decision making in resource limited households.

To address the gaps in existing literature, we developed “Eat My ABCs,” a school-based mindful eating program, designed to cultivate mindful eating skills among preschoolers as a strategy to prevent obesity [21]. A one-group pragmatic trial was conducted to evaluate the intervention’s effects on obesity prevention, household food insecurity, eating behaviors, and home eating environment. The findings showed significant improvements in the home eating environment and a reduction in household food insecurity. Furthermore, a clinically meaningful decrease in BMI z-score (0.20) among overweight or obese preschoolers was observed [21].

To optimize intervention effects and guide future implementation efforts, it is essential to comprehensively understand the acceptability and satisfaction of key stakeholders, in this case caregivers and teachers [22]. Understanding their perspectives will provide valuable insights to refine the intervention, improve its feasibility, and enhance its scalability across diverse early childhood settings. Therefore, this mixed methods study aimed to explore the acceptability and satisfaction of the “Eat My ABCs” program among both caregivers and teachers.

## 2. Materials and Methods

Study Design and Setting: A convergent parallel mixed methods analysis was conducted to evaluate teachers’ and caregivers’ experiences with the “Eat My ABCs” mindful eating program. The program was conducted from 2023 to 2024 in 26 Head Start daycare classes located in rural Michigan, U.S. The goal of the one-group quasi-experimental trial was to examine the program’s effects on preschoolers’ anthropometrics, dietary intake, and household food insecurity.

Study Participants’ Selection Criteria: Caregivers, defined as the preschooler’s primary caretaker, and their preschoolers were recruited non-randomly through study flyers distributed by daycare teachers. Interested caregivers were instructed to scan a QR code or follow a survey link to complete the enrollment and pre-program survey via Qualtrics. Preschoolers (ages 3–5 years) and their caregivers without food restrictions that would prevent dietary changes were eligible to participate. At the conclusion of the program, caregivers completed an online post-program survey containing quantitative evaluation questions. Teachers also completed a post-program evaluation survey. Semi-structured interviews were conducted with a purposefully selected group of caregivers and teachers to gather qualitative evaluation data. The purposive sampling was stratified by each class’s enrollment rate: low (≤5 per class), moderate (6–9 per class), and high (≥10 per class). Within each enrollment stratum, four classes were purposefully selected to represent the range of enrollment levels, and classroom teachers were invited to participate in interviews. For each selected class, approximately three caregivers were conveniently recruited to complete interviews. Both surveys and semi-structured interview guides are included in Supplemental File S1.

Ethical Considerations: Prior to data collection, caregiver consent was obtained. Teachers provided consent before completing surveys and interviews. This study was approved by the Institutional Review Board at Michigan State University.

Intervention: The “Eat My ABCs” program is a 14-week initiative designed to foster mindful, healthy, and lifelong eating habits in preschoolers. Each week, preschoolers were introduced to and tasted two of 26 fruits and vegetables, following the alphabet to incorporate school readiness learning. During the taste-test activity, they engaged in a mindful eating exercise using their five senses (touch, sight, hear, smell, and taste). After each lesson and mindful eating practice, preschoolers created a letter using fruit and vegetable stickers to express what they liked and wanted to try at home. These letters were sent home to caregivers to encourage continued mindful eating practices at home. All children enrolled in the participating classes took part in the lessons, regardless of whether they were eligible for or enrolled in the study. The lessons were taught by trained daycare teachers after completing a comprehensive four-hour training on childhood obesity prevention, mindful eating principles, and program implementation. Teachers were provided with all necessary materials, including food preparation supplies, a curriculum book, picture cards of the featured fruits and vegetables, and lesson videos. To further support healthy eating at home, every family in the participating daycare classes received two MyPlate plates and a cookbook featuring family- and kid-friendly recipes and cooking strategies to make nutritious eating more enjoyable.

Quantitative data collection: Separate post-intervention surveys containing program evaluation questions were administered to caregivers and teachers. The caregiver survey contained 11 close-ended questions and one open-ended question (see Supplemental File S1). These evaluation questions assessed caregivers’ acceptance and satisfaction with the overall program, program cookbook, and child letters. Response options varied by question. For example, one question stated, “The program has improved my families’ eating behavior,” with response options of strongly disagree, disagree, no opinion, agree, and strongly agree. Caregivers received compensation of a $20 e-gift card for completing the post-program survey containing these evaluation questions. The teacher survey consisted of 11 close-ended and three open-ended questions (see Supplemental File S1). The close-ended questions assessed teachers’ evaluation and satisfaction with the child program, with response choices of strongly disagree, disagree, agree, and strongly agree. One example question is, “Children actively engaged in healthy eating learning.” The three open-ended questions focused on teachers’ suggestions and the support needed to continue implementing the program in their classrooms. Each teacher received a $10 e-gift card for completing the survey. 

Qualitative data collection: When completing the online surveys, caregivers and teachers were asked whether they would be willing to participate in a 30 min individual interview at the end of the program. To ensure information was gathered on the differences between classes with varying enrollment, those who selected “Yes” were purposefully sampled based on classroom enrollment levels until data saturation was reached. Four caregivers and three teachers from high-enrollment classes (≥10 enrolled/class), 10 caregivers and four teachers from moderate-enrollment classes (6–9 enrolled/class), and three caregivers and one teacher from low-enrollment classes (≤5 enrolled/class) completed the individual interview via Zoom or telephone following the study’s semi-structured interview guides (see Supplemental File S1). During the interviews, caregivers and teachers provided feedback on the overall program, recruitment flyer, child program and letters, and any additional comments. Interviews continued until reaching data saturation, determined when no more novel information was gathered from the interviews. Audio recordings were transcribed using a free web-based transcription tool, and two trained research assistants independently reviewed all transcripts for accuracy.

Data analysis: Quantitative data cleaning and analyses were performed using SPSS Statistics Version 28. Means and standard deviations were calculated to summarize continuous variables, and frequencies and percentages were used to describe categorical variables. Chi-square tests were conducted to examine the association between family income level and caregiver evaluation survey responses. Qualitative data was analyzed using thematic analysis to systematically identify, analyze, and interpret patterns of meaning (themes) within the data [23]. We followed the six phases of thematic analysis [24]: (a) familiarize ourselves with the data, (b) generate initial codes, (c) search for themes, (d) review themes, (e) define and name themes, and (f) produce the report. JL developed an initial coding scheme based on the study’s semi-structured interview guides and informed by previous literature using a deductive approach [25]. ST and RB then independently applied the coding scheme deductively to the transcripts to ensure inter-rater reliability, meeting regularly to discuss any dissimilarities and refine coding results until reaching consensus. Any new codes that inductively emerged during this process were brought to JL for further refinement of the initial coding scheme. HL subsequently reviewed and synthesized the finalized themes, integrating them with quantitative data to provide a comprehensive, mixed methods interpretation of the study results.

## 3. Results

### 3.1. Demographics

All 402 preschoolers enrolled in the 26 participating classrooms received the intervention and data were collected from 200 (49.8%) preschoolers and 192 consented caregivers at baseline. At post-intervention, 153 caregivers (79.7%) completed the survey (see Table 1). Out of the 192 consented caregivers, 39 (20.3%) were contacted to complete an interview, and 17 (43.6%) completed the interview, reaching data saturation. Four caregivers scheduled an interview but did not answer the phone at the scheduled time and could not be reached to reschedule. The remaining 18 did not respond to the interview request. All 17 caregivers interviewed were white, non-Hispanic, and predominantly female (see Table 1). Their mean age was around 34 years, with the majority being married (70.6%) and unemployed (58.8%). The average caregiver interview length was 14 min.

Twenty-three (88.5%) of the 26 participating teachers completed the post-program demographic and evaluation survey. They were mostly female (22, 95.7%) with a mean age of 38 years. Eleven (47.8%) had a family annual income between $30,000 to $49,999, and 16 (69.6%) had a bachelor’s degree or higher. Their experience working in Head Start programs ranged from 0.5 to 30 years, with an average of 9.5 years (SD = 6.63). Of the 16 teachers who consented to be interviewed, 12 (75.0%) were contacted before data saturation was reached, and 8 (66.7%) completed interviews. The average interview length was 27 min. The two groups (interviewed vs. non-interviewed) were largely similar (see Table 2).

### 3.2. Thematic Results

Several key themes emerged from the quantitative and qualitative data, reflecting participants’ experiences and perceptions of the program. These themes included: (1) positive overall program evaluation, (2) strong future demand and participation, (3) improved food preferences and health behaviors, (4) enhanced caregiver-child communication, (4) implementation challenges, (6) value of teacher training, and (7) recruitment strategies.

#### 3.2.1. Positive Overall Program Evaluation

Both the qualitative and quantitative data (see Table 3 and Table 4) overwhelmingly indicate positive perceptions of the program. High levels of satisfaction were reported by both caregivers (88.9%) and teachers (87.0%). Furthermore, 19 teachers (82.6%) believed the curriculum helped children eat more fruit and vegetables, and 102 caregivers (66.7%) agreed that the program improved their family’s overall health.

These positive perceptions were further expressed during the caregiver qualitative interviews. During interviews, 16 of the 17 caregivers (94.1%) expressed favorable views of the program, with the remaining one caregiver reporting unfamiliarity with the program. Many caregivers shared that their children were enthusiastic about trying new foods. For example, one caregiver shared, “I think that he loved it. He would come home every day, let us know what letter and what he ate with the program, if he liked it, if he didn’t like it.” Another stated, “He’s come home super excited about learning the new food groups and wanting to try them at home.” A few caregivers noted that this program provided their children opportunities to try foods that would otherwise be inaccessible due to cost, “But like the dragon fruit, those are like four dollars, five dollars apiece. So, I’ve never even tried a dragon fruit myself to know what it tastes like. So, for them to be able to try something that I don’t have the means to get often or at all is great for her.”

Similarly, all interviewed teachers viewed the program as beneficial to the classroom. They described it as a “positive experience,” and something they “looked forward to doing every week.” One teacher shared, “We really enjoyed the program, my whole class did, all the kiddos.” Another added, “We were able to tie it into our studies even more on top of like letters and stuff like that.”

#### 3.2.2. Strong Future Demand and Participation

The program was widely viewed as easy to implement and participate in, suggesting strong potential for continued engagement by future teachers and families. This was well demonstrated in the caregiver evaluation survey: 150 caregivers (98.7%) agreed that they would recommend the program to others, and 147 (96.7%) stated they would participate again. Additionally, nearly half of the caregivers (47.4%) noted that the program required little to no effort to engage in, as it did not demand active participation from caregivers.

Of the 23 teachers who completed the post-program survey, 87.0% indicated they would continue teaching the curriculum in the future. One interviewed teacher said, “I thought it was a good program, and I would absolutely do it again.” When asked about the support needed to sustain the program, four interviewed teachers (50.0%) stated they would not require any additional resources. Others highlighted the importance of having access to the child letters, “would definitely need access to the questionnaires for the kids to fill out that I liked it” and noted that “Funding for the fruit and vegetables would be challenging.”

#### 3.2.3. Improved Food Preferences and Health Behaviors

Surveyed teachers overwhelmingly agreed that the curriculum successfully encouraged children to eat more fruits and vegetables (*n* = 19, 82.6%) and increased their healthy eating skills (*n* = 21, 91.3%). Among the eight teachers interviewed, seven (87.5%) described that their classroom children were more willing to try new foods, even outside of the program. Teachers shared examples such as, “Some kids were picky eaters and refused to try anything. Now kids will try things and like it.” Another noted, “They picked up on things and knew if it was good or bad for them. They started saying things like ‘apple is good for your brain.’” Additionally, one teacher recounted how the mindfulness approach enhanced food exploration, “We did the ‘let’s smell it, put it on our nose, or put it on our lips, put it on our tongue.’ Sometimes they would at least get it to their tongue, some of them wouldn’t have done that before.”

Caregivers also noticed positive changes in children’s health behaviors at home. They specifically mentioned that their children were less picky and more willing to try new foods, “I just think it was a good way for him to eat things that he normally wouldn’t eat. We have very picky eaters. It was the edamame that he loved. Yeah, he would never eat edamame unless it was given to him in a way like this”, and “[child name] is eating a few different fruits now that she wouldn’t even touch, so I believe it’s working.” One was surprised that their child liked some of the fresh vegetables, “I thought it was nice to see different things he tried that we might not have tried at home, and some I’m surprised he actually liked, I think it was avocado.” Caregivers often attributed this willingness to peer influence, “It could help that she’s with her friends and they’re eating it”, “The kids being in the classroom around their peers kind of helps them try new things,” and “Her friends were all into it, so she was into it.”

In addition to child-level changes, many caregivers noticed improvements in their family’s overall eating behavior. The majority of surveyed caregivers (*n* = 102, 66.7%) agreed that the program improved their family’s health. Of the 17 caregivers interviewed, 15 (88.2%) mentioned observing changes in their child’s or family’s eating behaviors. For instance, one caregiver stated “Yeah, we eat healthier. She tried more things, so she wants to eat more healthier foods, and so we eat healthier.”

#### 3.2.4. Enhanced Caregiver–Child Communication

The child letters, half-sheet reflections completed at school by children using stickers, served as a valuable conversation starter between caregivers and their children about the program. Most surveyed caregivers (*n* = 124, 81.1%) agreed that the letters helped them better understand what their children were learning in school. Interviewed caregivers echoed the following, “The kids come home and show them to me… If it is something we haven’t tried yet, she’ll ask me to try it,” “When he comes home, he shows me that card and then he has so much to say about the food that he tried and if he liked it or if he didn’t like it,” and “The papers are helpful because they give us talking points when kids don’t remember.”

In addition, 57.2% of the surveyed caregivers reported that they used the letters to inform food shopping and meal preparation for their families. This was also demonstrated in the caregiver interviews. One caregiver said, “We’re actually incorporating a lot of the stuff that she likes [into our meals at home], and doing a lot more hands on shopping with her and she receives her own list and gets to pick out her own stuff, so it’s been a lot of fun for us.” Another stated, “We’ll have like a good conversation about what she liked about it and wanting to put it on the list for next time we do our grocery runs. She gets to pick out things that she recently just tried and liked at school.”

According to the teachers interviewed, children enjoyed completing the letters, especially using the fruit and vegetable stickers. They acknowledged that the letters were helpful for connecting with caregivers. However, six (75.0%) of the eight teachers interviewed felt the children did not fully grasp the purpose of the letters. While the activity supported fine motor skills, some teachers questioned the letters’ educational impact for children, stating, “Kids enjoyed it; however, not super effective, as they are just happy with putting stickers down and not realizing the connection of the sticker. I understand how it is helpful for the parents though.” Another shared, “Good fine motor skills, but they didn’t understand what they were doing.”

#### 3.2.5. Implementation Challenges

A few challenges or barriers to implementing the program were identified during teacher interviews. Four (50.0%) interviewed teachers mentioned difficulty procuring specific fruits and vegetables due to limited availability in local stores. Moreover, two teachers described challenges with children’s engagement or comprehension of the curriculum, “They’re just interested in eating the food and not so much the learning part,” and “I really think the only thing that was a little hard for them to understand was the nutritional information.”

#### 3.2.6. Value of Teacher Training

Teachers expressed appreciation for the training session provided before the start of the school year. They found it comprehensive, well-structured, and that it prepared them to implement the child program in their classroom. Interviewed teachers highlighted, “Great information in the training, I liked human examples of what lessons would look like,” and it was, “Really good. Very informational. It made me feel like I was prepared to do anything.” This sentiment was echoed in the survey, where 21 (91.3%) out of 23 teachers surveyed agreed that they felt confident teaching the curriculum lessons, suggesting the training effectively supported their preparation.

#### 3.2.7. Recruitment Strategies

Both caregivers and teachers were asked about recruitment strategies during interviews. All participants who recalled seeing the recruitment flyer agreed it was helpful and informative in explaining the program. When asked who should approach families about enrollment, a slight majority of interviewed teachers (*n* = 4, 50.0%) and caregivers (*n* = 7, 41.2%) believed teachers should take the lead. One teacher reasoned, “Teachers because we build that connection with families.” Similarly, a caregiver shared, “I think the teachers would be the best because they’re the ones who are more interactive with the kids and with the parents.” Conversely, one (12.5%) teacher and three (17.6%) caregivers felt researchers should approach families. They emphasized the importance of building a clear connection between the classroom activities and the research project, especially since caregiver involvement was minimal. As one caregiver explained, “I’d probably prefer that you guys did [the recruiting], because you guys know more about the program.” Finally, three teachers (37.5%) and six caregivers (35.3%) suggested a combination of both teachers and researchers would be ideal. They stated that teachers could help initiate conversations and that researchers could provide further information during classroom events or caregiver meetings.

## 4. Discussion

This study complements prior literature from previous iterations of this program demonstrating that caregivers and teachers of preschoolers consider mindfulness-based programs acceptable, satisfactory, and desired in these communities [25,26]. The caregiver satisfaction rate for the Eat My ABCs program (88.9%) was a little lower than that of a previous similar program that had a caregiver component (95.2%) [25]. In addition, the proportion of caregivers who reported buying and preparing food according to the children’s letters was slightly lower (52.2% vs. 86.9%). These differences may be attributed to the absence of a formal caregiver component in the Eat My ABCs program, which may have led caregivers to feel less involved and informed. In addition, in the previous program, researchers directly sent copies of the weekly letters to caregivers to ensure delivery. Whereas the Eat My ABCs program relied on teachers and children to deliver the letters, which may have resulted in inconsistent caregiver receipt. As research around mindful eating intervention programs in the school setting is lacking, there are not any acceptability or satisfaction analyses to directly compare these results to. More research is needed surrounding the benefits and satisfaction of mindful eating curriculum for young children in the daycare and school setting.

While the main finding of this study is the acceptability and satisfaction with the population, results may also shed light on underlying mechanisms through which school-based mindful eating interventions, such as Eat My ABCs, lead to positive changes in child eating behaviors [21]. One plausible and redocumented mechanism is peer influence and teacher modeling. A 2023 scoping review concluded that exposure to food modelling by peers and teachers in early childhood school settings influenced children’s eating behaviors [27]. Similarly, a 2024 review focusing on children aged 2–5 years old found that peer influence played an important role in shaping eating behaviors [28]. While peer influence was positive in the present case, it is important to note that it can also contribute to increased consumption or disordered eating [29]. The Social Cognitive Theory and its precursor Social Learning Theory, developed by Albert Bandura, provide a useful framework for understanding this mechanism. These theories propose that children learn health behaviors through observing others in their social environment [30,31]. In other words, children acquire new behaviors by watching and modeling peers, teachers, and caregivers. Given that eating habits are established at an early age and persist into adulthood [32,33], both childcare providers and caregivers must be mindful of peer influence and the modelling behavior they present.

In addition to teacher and peer influence, caregiver beliefs, attitudes, and feeding practices are known to shape preschoolers’ eating behaviors [34]. In the Eat My ABCs program, weekly child letters could have prompted caregiver–child conversations around healthy eating, possibly reinforcing program messages. This aligns well with the Theory of Planned Behavior, which posits that health behaviors are driven by behavioral intentions, shaped by attitudes, subjective norms, and perceived behavioral control [35]. This framework is particularly relevant to understanding how caregivers’ beliefs and attitudes influence feeding practices and, subsequentially, shape children’s eating behaviors [34]. By leveraging the combined influence of peers, teachers, and caregivers, this mindfulness-based program has the potential to promote lasting improvements in child eating behaviors across both school and home settings.

Another recurrent finding of this study was that caregivers and teachers consistently reported that the program helped children be more willing to try new foods and be less picky. This aligns with literature indicating that mindfulness-based interventions can reduce picky eating in children [36,37,38,39]. Introducing mindfulness at an early age may help establish a healthier lifelong relationship with food, underscoring the importance of incorporating mindful principles during early childhood. Although the impact on pickiness was not measured quantitively in this study, the recurring mention by both caregivers and teachers highlights its perceived relevance anecdotally. These findings suggest a promising direction for future interventions to explicitly address food pickiness and neophobia among children.

Several challenges to sustain programs like Eat My ABCs were identified, including procurement difficulties, limited funding for fresh foods, and continued access to program materials such as child letters and sticker templates. Some barriers are particularly difficult to overcome in resource-constrained settings. A promising way to address both procurement and cost barriers is to partner with local farms or greenhouses to supply early childhood centers with fresh fruits and vegetables at a discounted rate. For example, Michigan’s Ten Cents a Meal for Michigan’s Kids and Farms program provides state-funded grants to schools, early care and education centers, and other organizations to purchase Michigan-grown foods (https://www.tencentsmichigan.org/about (accessed on 1 July 2025)). This initiative embeds farm-to-school support within state policy, streamlining access to funds and facilitating partnerships between local producers and educational institutions. A similar national policy initiative could support Head Start centers serving low-income families and children in procuring affordable fresh produce while stimulating local agricultural economies. Additionally, embedding mindful eating curricula into early child education policy standards could help unlock sustained funding for food procurement. To address ongoing access to program materials, child letters, stickers, and other resources were preloaded onto tablets provided to each teacher at the start of this and subsequent programs. These tablets remain with teachers to support continued use and long-term implementation of the program beyond the study period.

Finally, caregivers and teachers offered helpful insights on effective strategies for recruiting families of low socioeconomic status into research studies. In this study, recruitment was entirely community-led: teachers engaged families directly, distributed flyers, and advocated for enrollment. Many caregivers and teachers rated this method of recruitment successful because of the existing trust between teachers and the families they serve. However, some interviewed teachers expressed a preference for recruitment information to also come from the research team, as researchers are better equipped to explain study details accurately. These perspectives suggest that the potentially most effective recruitment approach for this population may involve a dual approach: leveraging trusted community partners who have established relationships with families, while also incorporating face-to-face interactions with knowledgeable research staff. Combining this relational strategy with other well-supported methods such as employing culturally sensitive staff, maintaining regular contact with participants, and offering non-coercive incentives, may be particularly suited to low-income populations who traditionally require more resources and tailored strategies for successful recruitment [40,41,42,43].

Strengths and Limitations: Strengths of this study include the use of a convergent mixed-methods design, allowing triangulation of findings across quantitative and qualitative data sources from both caregivers and teachers. This multi-informant approach enhances the depth, credibility, and contextual understanding of the results. Additionally, the study intentionally targeted a vulnerable population, young children from low-income backgrounds, addressing critical health disparities during a sensitive developmental window. By focusing on an underserved group within real-world Head Start settings, the study strengthens both its public health relevance and external validity.

This study has a few limitations. First, the optional interview selection process may have introduced self-selection bias. Caregivers and teachers who agreed to be interviewed may have had particularly positive or negative experiences that influenced their decision to participate. Second, the racial and ethnic distribution of interviewed caregivers was not fully representative of the overall study sample, potentially leading to some sampling bias. Third, the study sample was limited to families of low socioeconomic status in rural settings, which may restrict the generalizability of this study’s results to more diverse or urban populations. Fourth, given the retrospective nature of the interviews, social desirability and recall bias may have influenced participants’ responses. Finally, due to the young age (3–5 years), children’s views of the program were not directly evaluated.

Future Research: As the trial applied a quasi-experimental design, future research should employ a randomized controlled trial (RCT) design with a larger and more diverse sample to strengthen causal inference and enhance generalizability. Future efforts may focus on examining the underlying mechanisms of change suggested by the study’s results. Effects on reducing food neophobia warrant rigorous evaluation, along with the sustainability of impacts on eating behaviors and weight trajectories over time. To further assess acceptability and satisfaction, subsequent research could implement the program across broader and more diverse early childhood settings and expand qualitative data collection to capture wider perspectives from caregivers, teachers, and administrators.

## 5. Conclusions

In summary, this convergent parallel mixed methods analysis revealed: (1) Eat My ABCs program was viewed acceptable and satisfactory by both caregivers and teachers; (2) the program’s effectiveness may be attributed to the interactive influences of peers, teachers, and caregivers; and (3) barriers such as classroom implementation challenges and recruitment logistics need to be addressed in future applications. Despite some limitations, integrating a mindful eating program into early childhood education settings is both feasible and needed to address pressing public health concerns such as poor eating habits, childhood obesity, and food insecurity. Additionally, participants reported anecdotal changes in children’s eating behaviors, influenced by peer modeling and improved caregiver–child communication. Future efforts should explore policy avenues to sustainably support programs like Eat My ABCs, particularly around procurement and funding, to ensure long-term success.

## Figures and Tables

**Table 1:** Caregiver demographics.

Caregiver (*n* = 192)	Interviewed (*n* = 17)		Not Interviewed (*n* = 175)		Completed Post-Intervention Survey (*n* = 153)		Did not Complete Post-Intervention Survey (*n* = 39)	
	Mean	SD	Mean	SD	Mean	SD	Mean	SD
Enrolled preschooler age (months, range 34–64)	47.60	7.20	47.70	6.64	47.33	6.52	49.41	6.17
Caregiver age (years, range 21–67)	33.82	8.78	32.03	7.22	32.59	7.74	30.64	5.48
Number of children in family ^1^	2.47	1.38	2.55	1.08	2.44	1.06	2.92	1.20
	*n*	%	*n*	%	*n*	%	*n*	%
Sex (male)	1	5.9	6	3.4	4	2.6	3	7.7
Ethnicity (Hispanic)	0	0	9	5.1	7	4.6	2	5.1
Race								
White	17	100	158	90.3	140	91.5	35	89.7
Black or African American	0	0	2	1.1	2	1.3	0	0
American Indian or Alaskan Native	0	0	4	2.3	3	2.0	1	2.6
Multiracial	0	0	8	4.6	5	3.2	3	7.7
Other	0	0	3	1.7	3	2.0	0	0
Marital status								
Married/partnered	12	70.6	98	56.0	90	58.8	20	51.3
Separated/divorced/widowed	2	11.8	16	9.1	13	8.5	5	12.8
Single	3	17.6	61	34.9	50	32.7	14	35.9
Family annual income								
Under $20,000	3	17.6	64	36.6	49	32.0	18	46.2
$20,000–$29,999	9	53.0	36	20.6	37	24.2	8	20.5
$30,000–$49,999	4	23.5	48	27.4	41	26.8	11	28.2
$50,000 or above	1	5.9	27	15.4	26	17.0	2	5.1
Employment status								
Full Time	1	5.9	61	34.9	48	31.4	14	35.9
Part Time	6	35.3	41	23.4	39	25.5	7	17.9
No	10	58.8	73	41.7	66	43.1	18	46.2
Education level								
Less than high school graduate	0	0	15	8.6	11	7.2	4	10.3
High school graduate	7	41.1	83	47.4	71	46.4	19	48.7
Some college	6	35.3	48	27.4	42	27.4	12	30.8
Technical school or community college degree	2	11.8	15	8.6	15	9.8	2	5.1
Bachelor’s degree	1	5.9	10	5.7	9	5.9	2	5.1
Graduate or professional degree	1	5.9	4	2.3	5	3.3	0	0

^1^ 6 or more children in family were coded as 6.

**Table 2:** Teacher demographics (*n* = 23).

Demographic Variables	Interviewed (*n* = 6)		Not interviewed (*n* = 17)	
	Mean	SD	Mean	SD
Age (range 26–64)	37.17	7.39	38.94	10.61
Number of years working at Head Start (range 0.5–30)	7.83	4.17	10.09	7.33
	***n***	%	***n***	%
Sex				
Male	1	16.7	0	0
Female	5	83.3	17	100
Ethnicity (Non-Hispanic)	6	100	17	100
Race				
White	6	100	17	100
Marital status				
Married/partnered	6	100	12	70.6
Separated/divorced/widowed	0	0	2	11.8
Single	0	0	3	17.6
Family annual income				
$30,000–$49,999	1	16.7	10	58.8
$50,000 or above	5	83.3	7	41.2
Education level				
High school graduate	0	0	1	5.9
Some college	1	16.7	0	0
Technical school or community college degree	0	0	5	29.4
Bachelor’s degree	3	50.0	10	58.8
Graduate or professional degree	2	33.3	1	5.9

**Table 3:** Summary of caregivers’ quantitative evaluations (*n* = 153) ^1^.

Survey Items	Response Options	*n*	%
How satisfied are you with the program?	Very Dissatisfied, Dissatisfied	5	3.3
	No Opinion	12	7.8
	Satisfied, Very Satisfied	136	88.9
How acceptable is the program to you?	Completely unacceptable, unacceptable	0	0
	No opinion	8	5.2
	Acceptable, completely acceptable	145	94.8
The program has improved my family’s health.	Strongly disagree, disagree	5	3.3
	No opinion,	46	30.1
	Agree, strongly agree	102	66.7
How much effort did it take to engage in the program? (*n* = 152)	No effort at all, a little effort	72	47.4
	No opinion	30	19.7
	Some effort, a lot of effort	50	32.9
How often does your family use the cookbook provided? (ONE answer ONLY) (*n* = 152)	Not at all (never used)	28	18.4
	Rarely (1 time a week)	48	31.6
	Sometimes (2 times a week)	56	36.9
	Often (3 or more times a week)	14	9.2
	Very often (every day)	6	3.9
Would you have liked to receive something other than a cookbook to help you cook healthy foods? (*n* = 152)	Yes	35	23
	No	117	77
The weekly letters made by my child have helped me to know about my child’s learning in school.	Strongly disagree, Disagree	4	2.6
	No opinion	25	16.3
	Agree, Strongly Agree	124	81.1
I bought and prepared food for my child according to the child’s letters. (*n* = 152)	Strongly disagree, Disagree	17	11.2
	No opinion	48	31.6
	Agree, Strongly agree	87	57.2
Would you recommend the program to other parents you know? (*n* = 152)	Yes	150	98.7
	No	2	1.3
Would you participate in our program again if given the chance? (*n* = 152)	Yes, I think so	147	96.7
	No, I do not think so	5	3.3

^1^ 39 (20.3%) out of 192 caregivers did not complete the post-intervention evaluation survey. Chi-square analyses indicated no statistically significant association between income level and caregiver evaluation responses (*p* = 0.069–0.965).

**Table 4:** Summary of Teachers’ Quantitative Evaluations (*n* = 23).

Survey Item	*n* (%)			
	Agree	Strong Agree	No opinion	Disagree, Strong Disagree
Curriculum				
1. The curriculum meets my expectations.	14 (60.9)	7 (30.4)	0 (0)	2 (8.7)
2. The curriculum content is informative.	12 (52.2)	10 (43.5)	0 (0)	1 (4.3)
3. The curriculum content is age appropriate for preschoolers.	10 (43.5)	7 (30.4)	1 (4.3)	5 (21.8)
4. The curriculum instructions are easy to understand.	10 (43.5)	12 (52.2)	0 (0)	1 (4.3)
5. I am confident that I can teach the lessons in the curriculum.	8 (34.8)	13 (56.5)	1 (4.3)	1 (4.3)
6. The curriculum session length (20 min) is appropriate for preschoolers.	9 (39.1)	7 (30.4)	2 (8.7)	5 (21.8)
7. The curriculum increases children’s knowledge and skills on mindful eating.	9 (39.1)	12 (52.2)	1 (4.3)	1 (4.3)
8. The curriculum helps children eat more fruits and vegetables.	10 (43.5)	9 (39.1)	1 (4.3)	3 (13.0)
9. I plan to continue teaching the curriculum in the future.	9 (39.1)	11 (47.8)	3 (13.0)	0 (0)
10. Overall, I am satisfied with the curriculum.	6 (26.1)	14 (60.9)	3 (13.0)	0 (0)
Child Experience.				
11. Child actively engaged in the mindful eating learning.	12 (52.2)	10 (43.5)	1 (4.3)	0 (0)

Note: Percentages may not sum to 100.0% due to rounding.

## Data Availability

The data presented in this study are available upon reasonable request from the Principal Investigator, Dr. Jiying Ling (lingjiyi@msu.edu). The data are not publicly available due to privacy and ethical considerations.

## Supplementary Materials

The following supporting information can be downloaded at: https://www.mdpi.com/article/10.3390/nu18071103/s1, File S1: Evaluation Survey Instruments and Interview Semi-Structured Guides.

## Abbreviations

The following abbreviations are used in this manuscript:

BMI	Body Mass Index
SPSS	Statistical Package for the Social Sciences
RCT	Randomized Controlled Trial
U.S.	United States

## References

[1] Tester J.M., Rosas L.G., Leung C.W. (2020). Food insecurity and pediatric obesity: A double whammy in the era of COVID-19. Curr. Obes. Rep..

[2] Carvajal-Aldaz D., Cucalon G., Ordonez C. (2022). Food insecurity as a risk factor for obesity: A review. Front. Nutr..

[3] St Pierre C., Ver Ploeg M., Dietz W.H., Pryor S., Jakazi C.S., Layman E., Noymer D., Coughtrey-Davenport T., Sacheck J.M. (2022). Food insecurity and childhood obesity: A systematic review. Pediatrics.

[4] Stierman B., Afful J., Carroll M.D., Chen T.C., Davy O., Fink S., Fryar C.D., Gu Q., Hales C.M., Hughes J.P. (2021). National Health and Nutrition Examination Survey 2017-March 2020 prepandemic data files-development of files and prevalence estimates for selected health outcomes. National Health Statistics Reports.

[5] Liu M., Inoue K., Boyd A., Aggarwal R., Marinacci L.X., Wadhera R.K. (2025). Obesity Prevalence Among Children and Adolescents in the United States, 2011 to 2023. Ann. Intern. Med..

[6] National Center for Health Statistics NCHS Data Query System: Obesity in Children, Measured. https://nchsdata.cdc.gov/DQS/?topic=obesity-in-children-measured&subtopic=&group=age-group&subgroup=25-years%2C611-years%2C1219-years&range=&estimate=.

[7] U.S. Department of Agriculture ERS Household Food Security in the U.S. in 2022. https://www.ers.usda.gov/publications/pub-details?pubid=107702.

[8] Metallinos-Katsaras E., Must A., Gorman K. (2012). A Longitudinal Study of Food Insecurity on Obesity in Preschool Children. J. Acad. Nutr. Diet..

[9] Chou Y.C., Cheng F.S., Weng S.H., Yen Y.F., Hu H.Y. (2024). Impact of household income on the risk of overweight and obesity over time among preschool-aged children: A population-based cohort study. BMC Public Health.

[10] Rogers R., Eagle T.F., Sheetz A., Woodward A., Leibowitz R., Song M., Sylvester R., Corriveau N., Kline-Rogers E., Jiang Q. (2015). The relationship between childhood obesity, low socioeconomic status, and race/ethnicity: Lessons from massachusetts. Child. Obes..

[11] Aris I.M., Wu A.J., Lin P.-I.D., Zhang M., Farid H., Hedderson M.M., Zhu Y., Ferrara A., Chehab R.F., Barrett E.S. (2024). Neighborhood food access in early life and trajectories of child body mass index and obesity. JAMA Pediatr..

[12] Hashemzadeh M., Akhlaghi M., Nabizadeh K., Kazemi A., Miri H.H. (2025). Maternal nutrition literacy and childhood obesity in food-insecure and secure households. Sci. Rep..

[13] Tester J.M., Xiao L., Tinajero-Deck L., Juarez L., Rosas L.G. (2022). Food insecurity influences weight trajectory in children with obesity. Child. Obes..

[14] Tapper K. (2022). Mindful eating: What we know so far. Nutr. Bull..

[15] de Lara Perez B., Delgado-Rios M. (2022). Mindfulness-based programs for the prevention of childhood obesity: A systematic review. Appetite.

[16] Warren J.M., Smith N., Ashwell M. (2017). A structured literature review on the role of mindfulness, mindful eating and intuitive eating in changing eating behaviours: Effectiveness and associated potential mechanisms. Nutr. Res. Rev..

[17] Seguias L., Ferriday D., Hinton E.C., McCaw T., Tapper K. (2025). Mindful eating and food intake: Effects and mechanisms of action. J. Exp. Psychol. Appl..

[18] Artiles R.F., Staub K., Aldakak L., Eppenberger P., Rühli F., Bender N. (2019). Mindful eating and common diet programs lower body weight similarly: Systematic review and meta-analysis. Obes. Rev..

[19] Omiwole M., Richardson C., Huniewicz P., Dettmer E., Paslakis G. (2019). Review of mindfulness-related interventions to modify eating behaviors in adolescents. Nutrients.

[20] Rasmussen E.B., Rodriguez L.R., Pemberton S. (2022). Acute and enduring effects of mindful eating on delay and probability discounting for food and money in food-insecure women. Mindfulness.

[21] Ling J., Zahry N., Chen S., Xie Y., Lalonde H., Robbins L.B., Kerver J.M. (2025). A mindful eating program improved home eating environment and reduced food insecurity among rural economically marginalized families. Crit. Public Health.

[22] Nastasi B.K., Varjas K., Schensul S.L., Silva K.T., Schensul J.J., Ratnayake P. (2000). The Participatory Intervention Model: A framework for conceptualizing and promoting intervention acceptability. Sch. Psychol. Q..

[23] Clarke V., Braun V. (2017). Thematic analysis. J. Posit. Psychol..

[24] Braun V., Clarke V. (2006). Using thematic analysis in psychology. Qual. Res. Psychol..

[25] Chen S., Ling J., Buhlman R., Tadavich S., Kao T.-S.A. (2025). Acceptability and satisfaction of a mindfulness-based healthy eating and stress management program targeting economically marginalized families in a pilot trial. J. Pediatr. Psychol..

[26] Ling J., Chen S., Zhang N., Robbins L.B., Kerver J.M. (2024). Happy Family, Healthy Kids: A healthy eating and stress management program in low-income parent–preschooler dyads. Nurs. Res..

[27] Willemsen A., Wiggins S., Cromdal J. (2023). Young Children’s mealtimes and eating practices in early childhood education and care: A scoping review of 30 years of research from 1990 to 2020. Educ. Res. Rev..

[28] Street S., Simoncini K., Byrne R. (2024). Peer influence on eating behaviour in early childhood: A scoping review. Appetite.

[29] Salvy S.J., de la Haye K., Bowker J.C., Hermans R.C.J. (2012). Influence of peers and friends on children’s and adolescents’ eating and activity behaviors. Physiol. Behav..

[30] Bandura A. (1977). Social Learning Theory.

[31] Bandura A. (1986). Social Foundations of Thought and Action.

[32] Nicklaus S., Boggio V., Chabanet C., Issanchou S. (2004). A prospective study of food preferences in childhood. Food Qual. Prefer..

[33] De Cosmi V., Scaglioni S., Agostoni C. (2017). Early Taste Experiences and Later Food Choices. Nutrients.

[34] Oudat Q., Miller E.L., Couch S.C., Lee R.C., Bakas T. (2025). Understanding Caregivers’ Influence on Preschoolers’ Eating Behaviors: An Integrative Review Guided by the Theory of Planned Behavior. Children.

[35] Ajzen I. (1991). The theory of planned behavior. Organ. Behav. Human. Decis. Process..

[36] Bennett C., Copello A., Jones C., Blissett J. (2020). Children overcoming picky eating (COPE)—A cluster randomised controlled trial. Appetite.

[37] Kennedy A.E., Whiting S.W., Dixon M.R. (2014). Improving novel food choices in preschool children using acceptance and commitment therapy. J. Context Behav. Sci..

[38] Hong P.Y., Hanson M.D., Lishner D.A., Kelso S.L., Steinert S.W. (2018). A field experiment examining mindfulness on eating enjoyment and behavior in children. Mindfulness.

[39] Schmitt S.A., Snyder F., Korucu I., Bryant L.M., Finders J.K. (2020). Pilot intervention enhances preschoolers’ self-regulation and food liking. J. Nutr. Educ. Behav..

[40] Cui Z.H., Truesdale K.P., Robinson T.N., Pemberton V., French S.A., Escarfuller J., Casey T.L., Hotop A.M., Matheson D., Pratt C.A. (2019). Recruitment strategies for predominantly low-income, multi-racial/ethnic children and parents to 3-year community-based intervention trials: Childhood Obesity Prevention and Treatment Research (COPTR) Consortium. Trials.

[41] Nicholson L.M., Schwirian P.M., Groner J.A. (2015). Recruitment and retention strategies in clinical studies with low-income and minority populations: Progress from 2004–2014. Contemp. Clin. Trials.

[42] Nicholson L.M., Schwirian P.M., Klein E.G., Skybo T., Murray-Johnson L., Eneli I., Boettner B., French G.M., Groner J.A. (2011). Recruitment and retention strategies in longitudinal clinical studies with low-income populations. Contemp. Clin. Trials.

[43] Brannon E.E., Kuhl E.S., Boles R.E., Aylward B.S., Ratcliff M.B., Valenzuela J.M., Johnson S.L., Powers S.W. (2013). Strategies for Recruitment and Retention of Families From Low-Income, Ethnic Minority Backgrounds in a Longitudinal Study of Caregiver Feeding and Child Weight. Child. Health Care.

