# Supplementary material for: Acceptability and Satisfaction of Eat My ABCs: A Mindful Eating Program for Preschoolers in Low-Income Families
Source: Nutrients. 2026 Mar 30;18(7):1103. doi: 10.3390/nu18071103 (PMC13074814; doi:10.3390/nu18071103)
Supplement: Supplementary file 1 [file nutrients-18-01103-s001.zip › nutrients-4173753-supplementary.pdf]

Supplemental File S1: Evaluation Survey Instruments and Interview Semi-Structured Interview Guides

Caregiver Post-Program Evaluation Survey

1. How satisfied are you with the program?
    - o Very dissatisfied
    - o Dissatisfied
    - o No opinion
    - o Satisfied
    - o Very satisfied

2. How acceptable is the program to you?
    - o Completely unacceptable
    - o Unacceptable
    - o No opinion
    - o Acceptable
    - o Completely acceptable

3. The program has improved my families' eating behavior.
    - o Strongly disagree
    - o Disagree
    - o No opinion
    - o Agree
    - o Strongly agree

4. How much effort did it take you to engage with the program?
    - o No effort at all
    - o A little effort
    - o No opinion
    - o Some effort
    - o A lot of effort

5. How often does your family use the cookbook provided? **(ONE answer ONLY)**
    - o Not at all (never used)
    - o Rarely (1 time a week)
    - o Sometimes (2 times a week)
    - o Often (3 or more times a week)
    - o Very often (every day)

6. Would you have liked to receive something other than a cookbook to help you cook healthy foods?

    Yes □ No

    If "YES", what would you have liked to receive to help you cook healthy foods?

    ____

7. The weekly letters made by my child have helped me to know about my child's learning in school.
    - o Strongly disagree
    - o Disagree
    - o No opinion
    - o Agree
    - o Strongly agree

8. I talked about the weekly letters with my child.
    - o Strongly disagree
    - o Disagree
    - o No opinion
    - o Agree
    - o Strongly agree

9. I bought and prepared food for my child according to the child letters.
    - o Strongly disagree
    - o Disagree
    - o No opinion
    - o Agree
    - o Strongly agree

10. How can we make the child letters more helpful?

____

____

11. Would you recommend the program to other parents you know?

Yes No

If "No", please tell us why.

____

____

12. Would you participate in our program again if given chance?
    - o No, I do not think so
    - o Yes, I think so

Supplemental File S1: Evaluation Survey Instruments and Interview Semi-Structured Interview Guides

Teacher Post-Program Evaluation Survey

Dear Head Start Teacher:

Thank you very much for your support in our *Eat My ABCs* program. Please fill out the following evaluation survey. Your feedback is very helpful and we appreciate your participation!

Please select ONE option for each statement below.

Overall EVALUATION on the child CURRICULUM					
	Strongly Disagree	Disagree	No Opinion	Agree	Strongly Agree
**1.** The curriculum meets my expectations.	O	O	O	O	O
**2.** The curriculum content is informative.	O	O	O	O	O
**3.** The curriculum content is age appropriate for Head Start children.	O	O	O	O	O
**4.** The curriculum instructions are easy to understand.	O	O	O	O	O
**5.** I am confident that I can teach the lessons in the curriculum.	O	O	O	O	O
**6.** The curriculum session length (20 minutes) is appropriate for Head Start children.	O	O	O	O	O
**7.** The curriculum increases children's knowledge and skills on healthy eating.	O	O	O	O	O
**8.** The curriculum helps children eat more fruits and vegetables.	O	O	O	O	O
**9.** I plan to continue teaching the curriculum in the future.	O	O	O	O	O
**10.** Overall, I am satisfied with the curriculum.	O	O	O	O	O
**11.** Children actively engaged in healthy eating learning.	O	O	O	O	O
**Please expand upon your assessment of any areas in which our program could improve. We welcome your suggestions and feedback.**					

	1. **What changes, if any, would you suggest for the program?**<br>____<br>2. **What is the need for programs like *Eat My ABCs* in Head Start facilities?**<br>____<br>____<br>3. **What support or assistance do you need in order to independently teach the curriculum in the future?**<br>____<br>____

Supplemental File S1: Evaluation Survey Instruments and Interview Semi-Structured Interview Guides

Caregiver Semi-Structured Interview Guide

Thanks for taking the time to share your opinions with us. Keep in mind that we're just as interested in negative comments as positive comments, and at times the negative comments are the most helpful. We're tape recording the session because we don't want to miss any of your comments. Information collected from this session will be used by the researchers at Michigan State University to help them plan future programs. Information will only be presented in presentations or publications in group form. No individual names or identifiable information will ever be listed with a participant's response.

OPENING QUESTION:

To start with, please tell me your overall experience participating in the program with your Head Start child.

PROBE: What are the activities you like most?

PROBE: What are the barriers/challenges that prevented you from participating in some activities?

RECRUITMENT:

What do you think about the recruitment flyer?
    Are the contents appropriate?
    What suggestions do you have to make the flyer more attractive?

Who do you think should approach you about the study?

When the flyers should be sent out to you?

How should the recruitment flyers be sent out to parents?

What recruitment strategies do you think will work best to recruit parents?

CHILD LETTER TO PARENTS:

What do you think about your child's letters?

How much has your child shared with you about the school program?

How does child's participation (child letters) influence what you are doing at home related to mindful eating and movement?

What can we do to make the child letters more effective?

Any comments on the school program for your child?

ENDING QUESTION:

How does the program in general influence your family's eating behavior?

Before we end our discussion, are there other thoughts, comments, or suggestions that you would like to share with me to improve our children's overall health, or to help us successfully involve parents in this type of research study?

ENDING COMMENTS:

Thank you very much for agreeing to participate in this discussion today. We really appreciate your time to share with us your thoughts and ideas.

Supplemental File S1: Evaluation Survey Instruments and Interview Semi-Structured Interview Guides

Teacher Semi-Structured Interview Guide

Thanks for taking the time to share your opinions with us. Keep in mind that we're just as interested in negative comments as positive comments, and at times the negative comments are the most helpful. We're tape recording the session because we don't want to miss any of your comments. Information collected from this session will be used by the researchers at Michigan State University to help them plan future programs. Information will only be presented in presentations or publications in group form. No individual names or identifiable information will ever be listed with a participant's response.

OPENING QUESTION:

To start with, please tell me your classroom's overall experience participating in the program.

PROBE: What are the activities you like most?

PROBE: What are the barriers/challenges that prevented your classroom from participating?

How does the program in general influence your classroom children's eating behavior?

What changes have your observed in your classroom's children due to participating in the program?

RECRUITMENT:

What do you think about the recruitment flyer?
Are the contents appropriate?
What suggestions do you have to make the flyer more attractive?

Who do you think should approach parents about the study?

When the flyers should be sent out to parents?

How should the recruitment flyers be sent out to parents?

What questions did the parents ask about the study or recruitment flyer?

How can we facilitate the recruitment?

What recruitment strategies do you think will work best to recruit parents?

CHILD PROGRAM:

What do you think about the teacher training on delivering the program?

Supplemental File S1: Evaluation Survey Instruments and Interview Semi-Structured Interview Guides

How can we improve the teacher training? (format: face-to-face or virtual, location, training content, duration, time of the year)?

What do you think about the child "Eat My ABCs" curriculum? (content age appropriate, curriculum book easy to follow, lesson duration reasonable)

What are the challenges you have when teaching the lessons?

What are your suggestions to effectively teach mindful eating to young children?

What do you think about helping children to complete the weekly child's letters using stickers for parents?

How can we make the child letters more effective?

What support do you need to incorporate the program into your classroom daily routine?

ENDING QUESTION:

What other thoughts, comments, or suggestions you would like to share to improve our children's overall health, or to help us improve our program?

What health promotion programs do you think your classroom's children need to foster a healthy future?

What health promotion programs are you interested in participating along with your classroom's children?

ENDING COMMENTS:

Thank you very much for agreeing to participate in this discussion today. We really appreciate your time to share with us your thoughts and ideas.
